# A reflection on the role of women in Science, Dentistry and Brazilian Orthodontics

**DOI:** 10.1590/2177-6709.26.2.e21spe2

**Published:** 2021-04-30

**Authors:** Cátia Cardoso Abdo QUINTÃO, Luísa Schubach da Costa BARRETO, Luciane Macedo de MENEZES

**Affiliations:** 1,2 Universidade do Estado do Rio de Janeiro, Departamento de Ortodontia (Rio de Janeiro/RJ, Brazil).; 3 Pontifícia Universidade Católica do Rio Grande do Sul, Departamento de Ortodontia (Porto Alegre/RS, Brazil).

**Keywords:** Dentists women, Orthodontics, Education, History of females in Sciences, Leadership

## Abstract

**Introduction:**

This paper reviews the history of women scientists in the ‘Western world’, whilst highlighting the persistent socio-structural issues that have led to the hiding and masking of the participation of women in Science. Further, a reflection is made of the situation of Dentistry, specifically in the field of Orthodontics in Brazil. The difference between genders is discussed, with the intention to map the progress of women in management and leadership positions, in both the academic and professional fields.

**Description:**

In Brazil, within Dentistry and Orthodontics, despite being in a numerical majority, women are still underrepresented in the area of professional leadership. This is true for Research Groups and Research Productivity; an example being the relatively low authorship of publications in a Brazilian journal of Orthodontics. They are also underrepresented as lead presenters at professional meetings, whilst there are also few female Presidents of professional organizations and associations.

**Conclusion:**

Despite being in a numerical majority, it is also important that women act in a more co-ordinated and consistent manner to achieve greater representation in these areas. The necessary changes in the structure in order to achieve this are not only of women and for women, but they must also involve the whole of society so that leadership, rights and duties are equally distributed between the genders.

## RESUMO

**Objetivo:**

Este estudo objetivou resgatar a história de mulheres cientistas nos principais períodos históricos do mundo ocidental, para realçar a questão socioestrutural persistente que “invisibiliza” e mascara a participação das mulheres na Ciência. A partir disso, realizou-se uma reflexão sobre a situação da Odontologia, especificamente na área de Ortodontia no Brasil, no que tange à diferença de gêneros, com a finalidade de mapear a atuação das mulheres nos cargos de gestão e liderança nos âmbitos acadêmico e profissional.

**Descrição:**

No Brasil, na área de Odontologia e Ortodontia, apesar de serem maioria numérica, as mulheres ainda são minoria na liderança de Grupos de Pesquisa, Produtividade em Pesquisa, na autoria de artigos em um periódico nacional da área de Ortodontia, palestrantes de congressos e na presidência de Associações de Classe.

**Conclusão:**

Apesar de apresentar maioria numérica, é importante que as mulheres atuem de maneira mais consistente. As mudanças necessárias na estrutura não são apenas da mulher e para a mulher, mas devem envolver toda a sociedade para que direitos e deveres sejam distribuídos de forma igualitária entre os sexos.

## INTRODUCTION

Studies analyzing the participation of women in the history of Science are a relatively recent occurence.^1^ Certainly, women have actively contributed to the progress of Science, in several fields, in the various periods of Western history.^1^ ^,^ ^2^

Due to social structure and concepts imposed by society, their names were often not widely known. Commonly they remained in the shadow of a brother, husband or male co-worker who received the credit for the work. This explains, in part, why so few women have been recognized amongst the ranks of the great scientists. Certainly, historically, there was no shortage of women scientists, but many have been “forgotten”^2^. Since the research history has been predominantly recorded by men, women have been made almost “invisible”. This was an issue that was particularly identified after the 1960s. This problem also appears to have occurred in the academic area, where the female presence was largely restricted to a supporting role, as conditioned by the ideology and social structure of the time - it was part of the culture.^1^ However, latterly reviews historians have revealed that women did have a very relevant role in Science^3^. If one revisits history, women can be credited with many of the advances in early agriculture, over the period of 8000 to 4000 years BC (prehistoric period), see Table 1. Women can also be identified amongst the first pharmacologists, discovering, by observation, attempt and experimentation, the various therapeutic effects of plants. The knowledge accumulated over millennia constituted almost the entire pharmacopoeia until the advent of therapeutic chemistry.^2^

**Table 1:** History periods and events that landmarked them.

Period	Prehistory	Antique	Middle Ages	Modern Age	Contemporary Age
Dates	Until 4000 BC	From 4000 BC to 476	From 476 to 1453	From 1453 to 1789	From 1789 to the present day
Landmark events	Origin of the human species	Invention of writing	Fall of the Roman Empire	Fall of Constantinople	French Revolution

In Antique period, women carried an essential part of scientific and technical progress. Certain periods were marked by cults and legends, in which women played a remarkable role as goddesses and figures associated with alchemy and agriculture, which, not by chance, symbolized fertility. The first names of female ‘scientists’ recorded in history are from Assyria and Egypt. However, it was in Mesopotamia that women had some autonomy, with the possibility of owning their own lands, businesses whilst occupying important functions such as magistrates. However, the scientific work of ancient Greece was largely reported by the misogynist vision of Aristotle, for whom women were inferior on the spiritual plane, reporting that they had a smaller brain and even suggesting they had a different number of teeth in comparison with men. However, not all Greek philosophers were misogynistic in their outlook. Socrates and Plato accepted intellectual equality and pleaded for women to receive the same education as men. In the schools of Pythagoras (570-495 BC), which focused on the study of Mathematics, Astronomy, Natural Sciences and Philosophy, women were admitted. In Athens, Plato continued the Pythagorean tradition, accepting women in his classrooms, yet due to Athenian laws, they had to dress as men. Also, in Greek cities, women practiced Medicine, but over time they were limited to the practice of Gynecology. In Rome, women benefited from a relatively favorable status compared to Athenians, where since 450 BC the girls were reported to have received a basic education, learning to read, write and perform mathematics. Also, female doctors were considered equal to their male colleagues, a unique situation in history, which would only occur again in the 20^th^ century.^2^

**Figure 1:**
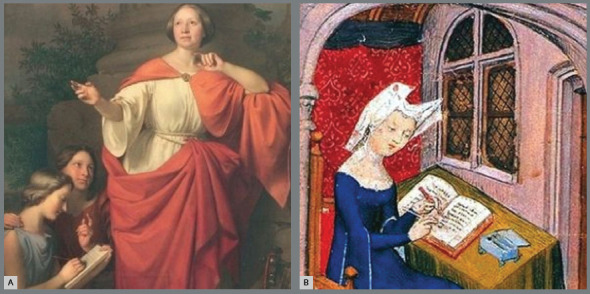
A) Representation of a female philosopher in ancient Greece. B) Illustration of Christine de Pizan, author of literary works that praise female education. Available at: https://mythicscribes.com/history/christine-de-pizan/.

In the middle ages, the repression of women in the intellectual field worsened with the growth of universities and their monopoly over knowledge. The rediscovery of Aristotle’s thoughts further impaired the situation. The growth of cities, formation of States and the strengthening of the Church led to the proliferation of urban schools in the 12^th^ century, the organization of universities in the 13^th^ century, and the creation and diversification of professions in the 14^th^ century. In the 13^th^ century, generally, women were left out of places where knowledge was diffused, i.e., schools and universities. This was a particular time for intellectuals, since knowledge became a source of social ascension, wealth and consideration. Misogyny was a structural component of the culture of the church and general social environment. Italy continued to be an exception to this rule.^2^ At the onset of the 15^th^ century, in France, the right to education had become the primary demand of women, based on the literary works of Christine de Pizan (*Querelle des Femmes* and *La Cité des Dames*, 1405)^4^, who put the question of women’s education at the center of this debate, against the accepted notion of their physical, intellectual and moral weakness (Fig 1). Christine stated that “if girls received the same education as boys and if they were methodically taught sciences, they would learn and understand the difficulties of all arts and all sciences as well as them” (men). For the first time, a woman dared to defy the general misogyny, giving strength to the debate, with participation of women and men from several European countries (France, England, Italy, Denmark).^5^

At the beginning of the Modern Age, a new market opened, grew and fragmented into dozens of new professions, thanks to the invention of the press in the middle of the 15^th^ century.^2^ The Scientific Revolution raised an unexpected enthusiasm for the whole subject and the related experimental method, with a multiplication of courses on these themes. Women actively participated in this movement, making important contributions, although it often generated criticism. The notion that the perceived defects assigned to women were due to the lack of education they received was gaining more credence and followers.^5^ After the 17^th^ century, with the advent of industrial capitalism, the social responsibility of men for production and women for reproduction^6^ became more evident. However, in France and England, the female society, aristocratic or bourgeois, fell in love with the sciences, discussed the latest inventions, learned mathematics and practiced Experimental Science. Women contributed significantly to the spread of new scientific and philosophical discoveries. Excluded from universities and academic institutions, women of the elites were assiduous in taking private courses, read a lot and created discussion meetings, which aided the intellectual diffusion of France abroad.^2^ After 1760, the need for an educational reform was recognized. Female education was then allowed, in the father’s home or in some institutions.^5^ However, outside the family environment, all intellectual activity was discouraged because it “contradicted the biological destiny of women”. Even so, in the period of the French Revolution, the philosopher Condorcet (1743-1794) unsuccessfully advocated mixed teaching based on the equality between genders. However, education plans continued to confine women to the household knowledge as necessary for family economics, in other words, reading, writing and some notions of arithmetic. For women from the wealthier classes, recreational arts such as Music, Singing and Dancing were included. Thus, excluded from all political activities, women could only achieve a primary education.^5^

Women from the 17^th^ and 18^th^ centuries participated in various scientific or technical activities. However, with a few exceptions, only a limited number managed to study in any depth. During the time of the Enlightenment (1715-1789), the important role of women in motherhood was identified, thus the role of the mother in the education and training of children became much more valued. Some women, that belonged to the noble or bourgeois classes, had the opportunity to receive a good education. However, generally they were relegated to the role of assistants or collaborators to well-known scientists. Notwithstanding, there were women who advocated their right to education and access to the same intellectual activities as men.^5^

In the Contemporary Age, after the French Revolution, Western democracies emerged, whose essence was equality. Philosophers of the time addressed the issue of gender by trying to explain, by reason and thought, a paradox: why human beings are equal to each other and unequal at the same time. The greatest philosophers, who based their works on democracy, the State of law, human rights and liberalism, were associated, almost without exception, with the idea that women were inferior to men, which would justify their submission to their father and/or husband. They argued that biological difference would explain the women’s inability to participate equally to men in political and intellectual life. However, in reality there was a need to move woman away from education, so that they would not become a threat to the perceived roles of men^2^. Thus, during this period, the vast majority of women were illiterate. Until the onset of the 19^th^ century, only a minority, from the aristocracy and upper bourgeoisie had access to further education, although these women could serve as example for many others.^2^ In the 19^th^ century, Science became more professional and also became a competitive activity, with the need for qualified people pursuing Science within the context of certain rules of conduct and hierarchy. Once again, women experienced great difficulties in entering the elitist and stratified institutions created, facing new problems, new forms of exclusion and consequently needing to adopt new strategies.^5^ Despite the advances in the intellectual condition of some women over the 17^th^ and 18^th^ centuries, there was a general stagnation in the 19^th^ century, based on recurring arguments that women were not made for Science, due to their nature and perceived roles within that society.^2^

In the 19^th^ century, significant changes occurred in the production process and organization of activities, with the consolidation of capitalism, which ended up expanding the need for female work.^6^ In the 20^th^ century, the advent of the two World Wars also facilitated the insertion of female labor, due to the need to replace the contingent of recruited male workers. However, women were exploited and subjected to subhuman working conditions, with long hours and receiving much lower wages than men. The ideological rationale for this, at the time, was that women had or should have someone to support them. However, socially, the woman was still responsible for the family dynamics and all duties related to it. Therefore, emancipation was only partial, resulting in the accumulation of double working hours, causing a significant disadvantage compared to men in the labor market.^6^

Some misogynistic views persist into the 21^st^ century. In 2005, Larry Summers, Dean of Harvard University, pointed out that the discrepancy could be related to the innate abilities of men compared to women, i.e., males would have a more naturally acquired aptitude for Science than females,^3^ ^,^ ^6^ in other words, biological differences might explain the reduced success of women in Science. Such views by certain academic leaders can only corroborate any existing prejudices and untruths that make difficulties for women pursuing a career in the Sciences.

Currently, women work in nearly all fields of professional activity, but there is a concentration in intermediate positions, whilst executive and management positions are still mostly occupied by men.^6^ The change in this scenario started in the second half of the 20^th^ century, with the increased need for human resources for Science. In addition, women’s liberation movements and the struggle for equal rights between men and women allowed them access to scientific education and careers traditionally occupied by men.^7^ Only in the second half of the 1970s and throughout the 1980s, the debate on equality and difference became the center of discussions. Cultural difference, female culture, female experience and the recognition of cultural gender diversity started to be discussed.^8^ Currently, in Western societies, men and women are moving away from stereotyped gender models and developing new forms of subjectivity, free from the divisions presented by society.^8^

## THE SCENARIO OF WOMEN IN BRAZIL IN THE 19^TH^ AND 20^TH^ CENTURIES

For nearly 450 years, there was a significant difference in schooling between Brazilian women and men, due to the prevailing social structure at the time. It is important to revisit the past to understand the historical context of the existing social structure and its influence on the role of women in society.

In the period between the end of the Second Empire (1840-1889) and the early 1920s, the city of Rio de Janeiro, the capital and most important city in Brazil at that time, was site to several movements, due to political and social dissatisfaction. The lower classes of the population were illiterate, with little political participation and no voting rights. The same was true for women, who were ultimately considered irrational, submissive and unable to discern public issues. Legally, they were subject to the father or husband, having no individual rights, freedom of conscience, thought, expression, religion, as well as mobility, work and management over patrimonial and heritage resources.^9^

During the Old Republic (1889 to 1930) women were considered different from men, not only in physical characteristics, but also in moral and psychological terms. Thus, women were seen as unstable and subjected to interventions from the environment, which could alter their normal development, hindering their primary function, which was considered to be reproduction. Cultural activities, education or work were seen as “harmful influences” for women. Medical professionals tried to highlight the differences between men and women, emphasizing their reproductive function^9^. Men were the holders of intelligence, reasoning and physical strength, with the power to change society, leading in Science and Politics. Women were responsible for motherhood and home. Thus, their education should emphasize hygiene, character and was based on the principles of moral, social and civic values, according to the Republican speech.^9^ The educational guidelines for boys and girls differed in content and they were not allowed to study together. According to the General Law of 1827, which regulated primary education in the country, girls only had access to the first level of education^9^. Over time, the need to educate future mothers, due to the project of modernization of the society and family, women gained greater access to education, increasing the need for more teachers for girls. Teaching would not subvert the role of women; rather, it could expand it. From then on, teaching began to be considered a typically female activity. The incompatibility of female professionalization with marriage and motherhood was one of the most persistent social constructions, and justified the lower wages offered to women.^9^ Speeches on women’s morals and bodies were based on religion, even after the advent of the Republic, with separation of Church and State, which became secular. Thus, the Medicine, Church and society legitimized a model in which marriage was seen as the social ideal, while work outside home was considered inappropriate for women.^9^

In various censuses, it was possible to observe the participation of women in the labor market. The 1890 Census provided little information on female labor, since it made no reference to activities related to the domestic field (such as washerwomen, seamstresses, embroiderers, cooks), which were mostly performed by poor women. In addition, in some categories of work there was no distinction by gender, such as in agriculture and industry. In the 1906 Census, it was found that 80.34% of working women were connected to domestic services. Also, in the 1920 Census, most women remained in the domestic service category (82.08%). In industry, the female participation continued to be higher in the clothing (62.18%) and textile sectors (39.26%). At that time, women were also present in the service sector (post offices and telegraphs - 31.92%) and then represented 81.20% of the total number of primary schoolteachers.^9^

In the Vargas Era (1930-1945) women were considered instruments to transform the country’s population, since they played the “female” functions that involved taking care of home, motherhood and caring for the family’s well-being. These functions were considered essential for the construction of a healthy, disciplined and productive population.^10^ At the same time, there was an awakening to the issues of female emancipation and the achievement of labor and political rights, such as the right to vote. As an example of these changes, separation is mentioned, which was instituted in the Civil Code in 1942, establishing the separation without dissolving the marriage bond, yet this condition was not socially well accepted.^11^ In 1943, the Brazilian law granted permission for married women to work outside home without the “express authorization of the husband”.^11^ The country was blooming, industrializing and in need of labor force, and women began to take up new work fronts. Conversely, their presence was advocated exclusively at home, as housewives and mothers.^10^ However, institutional and social changes continued to occur.

In the 1960s, the feminist movement gained strength. In 1962, twenty years after the introduction of separation, the Statute of Married Women came into force, which recognized her condition as one of “companion, consort, collaborator of the family’s responsibilities, responsible for ensuring its material and moral direction”. This was undoubtedly an advance in relation to the Civil Code of 1916, which considered women “incapable”.^11^ Also, after the 1960s, women in Brazil started to have access to more efficient contraceptive means (birth control pill, in 1962). Educational possibilities have also increased, with repercussions to the family relationships. In 1961, the Law of Brazilian Education Guidelines and Bases assured the equivalence of high school courses, allowing students in the teaching profession (“Escola Normal”) to compete for places in higher education.^11^ In the 1960s and 1970s, Brazilian women changed their values ​​and ideals. There was an increase in women’s participation in the labor market and also a struggle for growth and professional recognition. Women had greater access to formal education and achieved the right to decide whether to become a mother. In 1977, divorce was instituted and also the possibility of establishing other affective relationships, socially recognized. Thus, after the 1970s, women of middle and upper classes could envision a professional future for their daughters, earning their own money, with life horizons beyond marriage, while simultaneously they began to occupy a more egalitarian position in relation to the husband.^11^

In the 1980s, Brazilian women changed their role in the family, society and the labor market. The Federal Constitution of 1988 provided relevant achievements^11^ because it expanded individual and social rights and consolidated women’s citizenship in the public space and family life. This assured rights in the fields of health (including sexual and reproductive health); safety; education; land ownership and access to housing; work, income, social security and access to civil and political rights.^12^ In the last decades of the 20^th^ century and early 21^st^ century, women reached important parts of the labor market, achieved greater schooling, managed to expand control over their sexuality and fertility, but also increased their working hours. However, despite all advances in recent decades, inequality is still evident, especially when comparing average wages, which are about 30% lower compared to men.^11^ Despite persistent social differences between men and women, families tend to form a more egalitarian relationship between partners, since both contribute financially to the maintenance of home and its members. This change empowered women within their families, breaking the old cycle of dependence and subordination. Following the changes in society and contributing, in turn, to change society itself, the “modern conjugal family” as proposed in the first half of the 20^th^ century is no longer the predominant reference.^11^ New family arrangements have emerged (single parents, reunited families, homosexual relationships). There was a transformation of families, a drop in birth rate, an increase in marriages and remarriages of the most varied types. Family unions and bonds have emerged, which reflect the affective relationships, and the Brazilian conjugal society is driven by loving relationships and individual satisfaction.^11^

## THE SCENARIO OF DENTISTRY AND ORTHODONTICS IN BRAZIL

Until the onset of last century, it would be impossible to think of a scenario in which women would form a majority in the field of ​​Dentistry in Brazil. Even at the end of the 19^th^ century, professional practice was performed mainly by dentists not formally trained, due to the lack of Dentistry Courses.^13^ This occurred until establishment of the first course, officially created in Brazil by a decree of the Imperial Government, signed by D. Pedro II, on October 25, 1884.^14^ Thus, at the end of the 19^th^ century, there were three Dentistry Courses in Brazil: at the Federal University of Rio de Janeiro, Federal University of Bahia and Federal University of Rio Grande do Sul,^15^ which were constituted almost exclusively by men. Dental practice by women was rare until the end of the Empire. As in other countries, women working in this field were basically limited to daughters, wives or widows of dentists (lay women who had achieved the profession from another practitioner).^13^ Few women in this period entered regular courses.

The teaching of Dentistry in São Paulo began in 1902, with female students from the start. From 1903 to 1926, 221 women (19.23% of the total graduates) and 928 men graduated from the current School of Dentistry at the University of São Paulo.^13^ The care of ladies and children patients was an option for many female dentists. Some had their own offices and others shared spaces with family and colleagues.^13^ At that time, a movement to regulate the profession was initiated in São Paulo. Thus, to continue their activities, non-graduated dental practitioners should undergo a qualification exam to a commission of graduates. In this process, in the state of São Paulo, 172 men and nine women who were licensed dentists enrolled in the Health Service between 1900 and 1925.^13^

Dentistry courses in Brazil have had an exponential growth since their establishment^15^. Women were in a minority until the 1980s, when dental schools began to train more women than men, with increasing feminization of the profession.^13^ The increase in the number of women in undergraduate and graduate courses, either as students, professors or researchers, as well as their access as scholars of research programs, have undoubtedly contributed to the inclusion of woman in all areas of Science and Technology agencies.^12^

Until the middle of the 20^th^ century, there were 24 Dentistry courses in Brazil, half of them concentrated in the Southeast region.^15^ There was a more intense expansion in the number of Dentistry courses since 1961, with the regulation of the Law of Brazilian Education Guidelines and Bases, which increased educational opportunities, creating financial and legal support for the private sector in the field of education, whilst promoting a great expansion of private education network in the country.^15^ The so-called “Renewal of the Brazilian University” movement (1968) expanded the number of Dentistry courses, opening higher education to private institutions for profit, which led to a change in the standards of higher education. The Teaching-Research-Extension triad was weakened in some institutions, prioritizing only Teaching, opening the Education market to institutions with a business profile. This expansion led to a four-fold increase in the number of Dentistry courses in Brazil until 1996, when, among 104 existing courses, 60 were offered in private institutions.^15^

Currently, there are 544 authorized Dentistry courses in Brazil, being the country with the highest absolute number of Dentistry courses in the world, running the risk of collapse due to the abundance of dentists in the job market.^15^ The greatest concentration of courses is still in the Southeast region (36%), followed by the Northeast (29%), South (16%), Central West (10%) and North (9%).^15^

The disorganized expansion also occurred in postgraduate courses in the field of ​​Orthodontics: in the 1950s there were 2 accredited courses, and in 2009 there were 309 courses^16^ (representing 1/3 of all Dentistry Specialization courses in Brazil). Thus, Brazil has a total of 344,041 dentists registered in the Federal Dental Council, almost 10% of which, i.e., 30,266, are orthodontists/functional orthopedists, and almost 60% of these (18,066) are women.

Compared to other countries as the United States and Canada, the uncontrolled growth in the number of postgraduate courses in Orthodontics in Brazil is quite evident. The United States has 67 Postgraduate courses in Orthodontics and Canada has 6, accredited by the American Commission on Dental Accreditation (CODA) and the Commission on Dental Accreditation of Canada (CDAC), according to data obtained from the American Orthodontics Association (AAO)^17^ website (Table 2). Analyzing the ratio between number of inhabitants and number of courses in Orthodontics in United States, Canada and Brazil, it is observed that, for the former, this ratio is 4.97 million, for Canada 6.26 million, while for Brazil, in 2009, with a population of 193.9 million, this index would be 627.5 thousand inhabitants per postgraduate course in Orthodontics.

**Table 2:** Number and coordination (by gender) of Postgraduate Courses in Orthodontics in the United States and Canada. Source: American Association of Orthodontists^17^, 2020.

Country	Coordinator man	Coordinator woman	Total courses
USA	55 (81.0%)	13 (19.0%)	67
Canada	5 (83.4%)	1 (16.6%)	6

Orthodontics was the first dental specialty to be recognized as such in June 1900. Edward Hartley Angle was elected the first president of the American Society of Orthodontists. The first postgraduate courses in Orthodontics in North American universities emerged in 1922, at the Universities of New York and Columbia. In Brazil, the first specialization course in Orthodontics started in 1951, at São Paulo Dental Association (APCD), functioning until 1955.^18^ The first specialization course in Orthodontics in a Brazilian university was initiated in 1959 at the School of Dentistry at the Federal University of Rio de Janeiro (UFRJ). In 1974 the course was raised to the MSc level and, in 1981, the PhD course was initiated. In 1962, at the Piracicaba School of Dentistry (currently belonging to UNICAMP), the second specialist course in Orthodontics was initiated, recognized as a MSc degree in 1974, and a PhD degree in 1983. In 1966, the course in Orthodontics was established at the School of Dentistry of the University of São Paulo (USP), which in 1974 was accredited as a MSc. At Bauru School of Dentistry, University of São Paulo (FOB-USP), in 1973, the course in Orthodontics was started, with an MSc degree in 1981 and a PhD course was commenced in 1982 and recognized in 1989.^18^ Thus, in a time span of 14 years (1959-1973), there were four graduate courses in Orthodontics in Brazil.^18^ In the last decade (2010-2020), 534 specialization courses in Orthodontics were registered in Brazil, according to the Federal Dental Council,^19^ as shown in Table 3, showing the exaggerated increase in the number of postgraduate courses in Orthodontics in the country.

**Table 3:** Number of Orthodontists; Specialist Courses in Orthodontics registered in the CFO (2010 to 2020) and Courses Coordinators according to sex (Source: data extracted from material provided by CFO^19^ and total of orthodontists registered in the CFO by region^20^).

Region of Brazil	Number of orthodontics	Number of courses	Courses coordinated by men	Courses coordinated by women	Courses without coordinator registration
North	1,513	32	22	7	3
Northeast	2,564	64	40	21	3
Midwest	3,085	50	28	13	9
Southeast	14,850	252	161	74	17
South	7,272	136	107	23	6
Total	29,284	534	358	138	38

**Figure:**
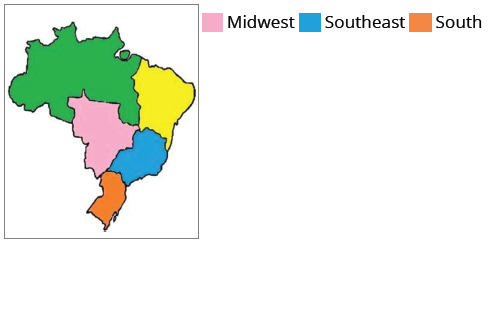

**Figure 2:**
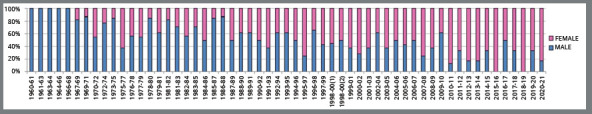
Distribution of Masters students in Orthodontics, UFRJ (from 1960 to 2020), by sex. Source: Archives of the XIX Alumni Meeting of UFRJ^21^, 2019.

**Figure 3:**
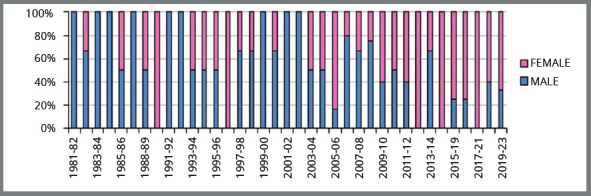
Distribution of PhD students in Orthodontics at UFRJ (1981 to 2020), by sex.

Source: Archives of the XIX Alumni Meeting of UFRJ^21^, 2019.

Analyzing the history of 60 years of the oldest postgraduate course in Orthodontics at a Brazilian university,^21^ the UFRJ, it was observed that, among all students graduating from the MSc Course (Fig 2), 52.7% are males and 47.3%, females. Until the fifth group (1966/1968) the composition was exclusively of men. From then on, until the 27^th^ group (1991/1993), there was predominance of males, except for the 11^th^ group (1975/1977). After 2010, there has been predominance of women in nearly all groups. Analyzing the total number of students graduating from the PhD Course at UFRJ (Fig 3), 52.2% are males and 47.8%, females. For both MSc and PhD courses, there is predominance of men at their beginning (1960s and 1980s), and currently a predominance of women.

The greater presence of women in the Academy in recent years may have facilitated the access and incorporation of women to the staff of Brazilian universities^7^. It seems that this phenomenon has been occurring with a gradual increase in the number of women in graduate courses. Due to their insertion in the academic career, many universities now have women in their staff. Thus, though still distant in relation to gender equality, they have come very close to men, i.e., the situation has been balancing. It should be noted that the insertion of women into the Academy began a long time after men. Therefore, when analyzing the progress and evolution of female participation over time, it is noticed that they are occupying spaces in an increasing and consistent manner.

Analyzing postgraduate courses in general, in 2019 women represented about 54% of PhD students in Brazil, indicating an increase of 10% in the last two decades. This number was similar to that of developed countries, such as the United States, where in 2017 women obtained 53% of PhD degrees awarded in the country. However, in Brazil, alike the rest of the world, this female participation varies a lot according to the field of ​​knowledge.^22^ Women form a majority in Life and Health Sciences (more than 60%), while in Mathematics and Computer Science they represent less than 25%.^22^

Analyzing the Graduate Courses in Dentistry in Brazil considered of an excellent standard (Scores 5, 6 and 7, Capes/2017, scores range from 1 to 7), it was observed that, among 22 courses, 14 are coordinated by women (63,64%) and 8 by men (3,36%) (Table 4). Possibly, the greater number of female students in undergraduate courses and graduate programs enabled their greater demand for positions of higher hierarchy in the system.^7^

**Table 4:** Coordinators of the Graduate Programs in Dentistry, scores 5, 6 and 7 (CAPES).

Score	Coordinator man	Coordinator woman	Total
5	6	7	13
6	2	4	6
7	0	3	3
Total	8 (36.36%)	14 (63.64%)	22

Source: Plataforma Sucupira; Accessed on: January 20, 2021. https://sucupira.capes.gov.br/sucupira/public/consultas/coleta/program/quantitativos/quantitativo Brasileiro.jsf?areaAv

With the high number of female orthodontists in Brazil, how are they distributed in management, prominence or leadership positions in the academic environment?

Considering the Coordination of Postgraduate Courses in Orthodontics in Brazil, specialist level, in the last decade, the Brazilian Federal Dental Council (CFO) registered 534 courses, among which 358 were coordinated by men and 138, by women (38 are unregistered) (Table 3).

Analyzing data presented by the AAO related to the American and the Canadian Postgraduate Courses in Orthodontics (Table 2), it was observed that, among 74 courses registered until 2020, 14 are coordinated by women (18.9%) and 60 (81.1%), by men.

**Figure 4:**
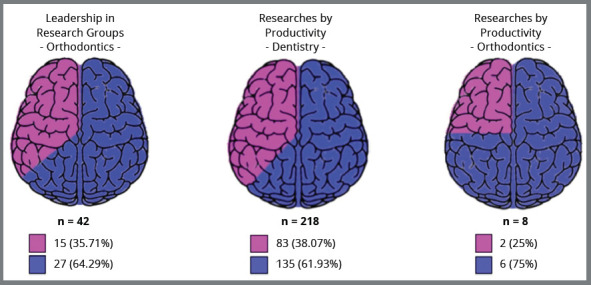
Leadership in Research Groups (Orthodontics) CNPq; and CNPq Productivity Researchers (Dentistry and Orthodontics) (pink color for women; blue color for men ). Source: CNPq ^23^, 2020.

At the beginning of the century, there was a growing trend in the percentage of women taking positions of researchers and leading figures in research groups, indicating a greater insertion of women in the system, not only as students, but in positions of greater recognition and higher hierarchical qualification.^7^ Statistics referring to the Brazilian National Council for Scientific and Technological Development (CNPq) research groups revealed a continuous process of approximation between the percentage of men and women researchers: in 1995, women represented 39% of researchers; in 2002, 46%; in 2010 parity was reached between genders.^7^ Specifically, in the field of ​​Orthodontics, there is still a smaller number of women leaders of Research Groups or Productivity Researchers of CNPq (Fig 4).

In 2003 it was already observed that the proportion of female scholars increased in different modalities, yet it decreased as the hierarchical level of the scholarship increased, indicating that part of the women who went through the first stages of qualification and training for scientific activities did not continue their careers or did not get peer recognition to achieve scholarships.^7^ At this time, women accounted for around 50% of all scholarship modalities (Scientific Initiation, MSc, PhD, Postdoctoral, Research Productivity and Technical Business Development). Only in the last two modalities, the percentage of women was lower than men.^12^ In 2019, women represented only 24% of Research Productivity Scholars, considering all fields of knowledge.^22^ In the case of Dentistry and also Orthodontics, there is still a predominance of males as Productivity Researchers (CNPq) (Fig 4). Why do women researchers, dentists, and orthodontists, with a high scientific productivity, fundraising capacity, a greater training in human resources and with a higher degree (fundamental requirements to coordinate Graduate Programs of Excellence in Dentistry at Brazilian Universities) not equal the number as Research Productivity Scholars or Leaders in Research Groups? Are women researchers, dentists and orthodontists actually less productive than men, or are there still socio-structural factors involved? 

Concerning the publication of scientific papers from all fields of knowledge, Brazilian women exceed the production of men.^22^ The impact of the work of men and women is comparable regarding the number of article citations. Between 2008 and 2012, women were already responsible for almost 70% of the total number of publications by Brazilian scientists, one of the highest proportions in the world.^22^

Considering the best ranked Orthodontics journal in Brazil (Dental Press Journal of Orthodontics, DPJO),^24^ which presents a high Impact Factor and Cite Score, the scientific production was evaluated in relation to the gender of authors, in the last 10 years. In the period from 2010 to 2020, evaluating all issues of the journal (6 per year), it was concluded that women presented a lower percentage than men in relation to authorship, both as first and last authors and also in relation to co-authorships (Table 5). The total number of authors (men and women) of DPJO in this decade was 3,238, being 1,956 (60.4%) men and 1,282 (39.5%) women, showing a lower percentage of women authors than revealed for the scientific production in general, namely 70% as mentioned above, for the period from 2008 to 2012. When considering the first author (who defines the executor of the research) the participation of women in the journal varied from 32.92% (2014) to 51.60% (2019). The last authorship (which defines the intellectual supervisor of the investigation) had female representation ranging from 25.58% (2016) to 49.12% (2015). Considering the total number of women in each article, they were also minority - between 36.59% (2013) to 45.58% (2019). Women reached a number close to men as first authors and in the total number of authorships in 2019, falling back in 2020.

**Table 5:** Publications from the Dental Press Journal of Orthodontics (2010 to 2020), with total authors per year, distribution of authors according to sex and authorship. Source: Dental Press Journal of Orthodontics^24^, 2020.

Journal	Year	Total articles	Total authors (men and women)	First Author	Total of co-authors	Last author	Total authors
DPJO	2020	66	221	26 (39.39%)	41 (37.61%)	14 (29.78%)	81 (36.65%)
	2019	62	204	32 (51.60%)	44 (44.00%)	17 (40.47%)	93 (45.58%)
	2018	61	217	26 (42.62%)	40 (38.46%)	16 (30.76%)	82 (37.78%)
	2017	70	237	26 (37.14%)	48 (40.67%)	16 (32.65%)	90 (37.97%)
	2016	62	218	21 (33.87%)	48 (42.47%)	11 (25.58%)	80 (36.69%)
	2015	82	279	28 (34.14%)	55 (39.28%)	28 (49.12%)	111 (39.78%)
	2014	82	269	27 (32.92%)	66 (51.56%)	20 (33.89%)	113 (42.00%)
	2013	114	440	46 (40.35%)	87 (37.02%)	28 (30.76%)	161 (36.59%)
	2012	127	471	55 (43.30%)	98 (41.35%)	43 (40.18%)	196 (41.61%)
	2011	89	337	39 (43.82%)	76 (44.18%)	27 (35.52%)	142 (42.13%)
	2010	95	345	43 (45.26%)	64 (37.20%)	26 (33.33%)	133 (38.55%)

The DPJO journal has already had 5 Editors since its onset in 1996, and since 2018 it has a female Chief Editor for the first time in its history.

Aiming to analyze the performance of women in Orthodontics Associations, data from the World Federation of Orthodontists^25^ and the Brazilian Orthodontic Association^26^ were consulted.

The World Federation of Orthodontists (WFO)^25^ was established on May 15, 1995, with the goal to develop the art and science of Orthodontics. It currently has 109 affiliated entities around the world.

The Brazilian Association of Orthodontics and Facial Orthopedics (ABOR)^26^ was established on January 25, 1994, with the aim of gathering regional associations, some of them much older as the Brazilian Orthodontic Society (1955) and Orthodontics Societies of the states of Paraná (1972), Rio Grande do Sul (1975), Espírito Santo (1985) and Minas Gerais (1985).^27^ In May 1995, the ABOR joined the WFO, then representing the Brazilian Orthodontics in the international scenario.

The WFO has had 6 presidents since its establishment, all men. Conversely, ABOR also had 6 presidents, being one woman. Currently, of the 22 regional offices of ABOR, 15 are directed by men and 7 by women (Table 6).

**Table 6:** Composition of WFO^25^ and ABOR^26^ (National and Regional Boards).

Entities	Total	Presidents	
		Men	Women
WFO (Presidents) (1995-2020)	6	6 (100%)	0 (0%)
ABOR - National Board (1994-2020)	6	5 (83.33)	1 (16.66%)
ABOR - Regional Offices (2020)	22	15 (68.18%)	7 (31.81%)

**Figure 5:**
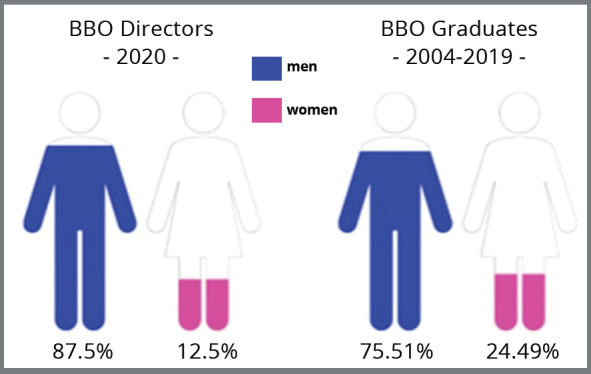
Composition of Directory and Graduates of the Brazilian Board of Orthodontics (BBO) according to sex. Source: BBO,^28^ 2020.

The Brazilian Board of Orthodontics (BBO)^28^ was created in 2002 due to the need to establish standards of clinical excellence to value the specialty. The evaluations started in 2004 and are made annually, based on the American Board of Orthodontics, with theoretical and clinical case examinations. Orthodontics was a pioneer in the Health area in Brazil to have an examination for the certification of professionals regarding clinical excellence. The proportion of BBO graduates and directors reveals a predominance of males, with 75.51% of graduates and 87.5% of directors composed of men (Fig 5).

The ABOR^26^ organizes an International Congress every two years with the participation of all 22 Regional Offices, the Brazilian Board of Orthodontics (BBO)^28^ and the Brazilian Group of Professors in Orthodontics and Pediatric Dentistry. All presidents of ABOR Congresses in the last 10 years (Table 7) were men. Only in 2022 the ABOR congress will have a woman as president.

**Table 7:** WFO^25^ and ABOR^26^ Congress Presidents (pink color for women; blue color for men).

Congress - year - location	Man	Woman
WFO - 2010 - Australia	1	
WFO - 2015 - London	1	
WFO - 2020 - Japan	1	
WFO - 2025 - Brazil		1
ABOR - 2011 - Belo Horizonte	1	
ABOR - 2013 - Natal	1	
ABOR - 2015 - Florianópolis	1	
ABOR - 2017 - Belém	1	
ABOR - 2019 - Rio de Janeiro	1	
ABOR - 2022 - Fortaleza		1

**Figure:**
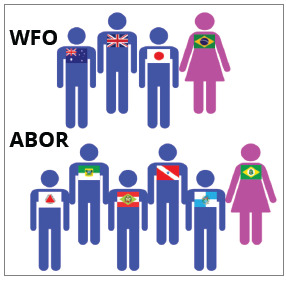

The female participation in prominent positions is the result of a personal effort by a few women to break a system structurally constituted by men. Thus, it is important to mention Professor Flavia Artese, from the State University of Rio de Janeiro, who was President of ABOR for two periods (2014-2018). She currently is the Chief-Editor of DPJO, the first woman to compose the Board of Directors of the College of Diplomates of the Brazilian Board of Orthodontics (CDBBO), will be president of the next WFO congress and is an “ambassador” of Brazilian Orthodontics in lectures all over the world. We mention her to represent the necessary recognition to pioneer women in Dentistry and Orthodontics in Brazil. So many others could be cited, since they open the doors to future generations, facing discrimination, restrictions and prejudices, and they have run an arduous way that served and serves as an example and inspiration for all.

Analyzing the scientific program of ABOR Congresses from 2011 to 2019, with data summarized in Table 8, there was predominance of male over female speakers (average 80.46% of men and 19.54% of women). This same pattern can be observed internationally, analyzing the WFO and its Congresses, held at every 5 years, with predominance of men as presidents and majority as speakers in the scientific programs (Tables 7 and 8). The last WFO congress organized in Japan (2020), held online due to the Coronavirus Pandemic, presented a program with a total of 99 speakers, being 77 men and 22 women (77.78% and 22.22%, respectively) (Table 8).

**Table 8:** Speakers at the WFO^25^ and ABOR^26^ Orthodontics congresses (pink color for women; blue color for men).

Congress - year	Total speakers	Speakers men	Speakers women
WFO - 2020	99	77 (77.78%)	22 (22.22%)
ABOR - 2011	79	67 (84.81%)	12 (15.18%)
ABOR - 2013	67	50 (74.62%)	17 (25.37%)
ABOR - 2015	85	71 (83.52%)	14 (16.47%)
ABOR - 2017	83	66 (79.51%)	17 (20.48%)
ABOR - 2019	139	111 (79.86%)	28 (20.14%)

**Figure:**
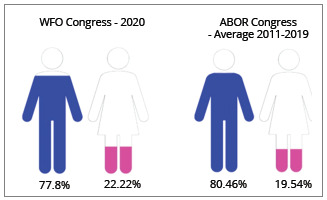

Why are women majority in the profession and a minority in their exposure? Does acting as a leader make men keep their peers in prominent positions, feeding a structural pattern that reinforces the women’s historical “invisibility”? Though unintentional, it could be a pattern of behavior, both for men and women, to consider this normal, as an outcome of the repetition of the current social structure. The way to achieve a more egalitarian society, with the same opportunities for men and women, begins with the manner through which the parents socialize and raise their children, boys and girls, without stereotypes or dream limitations and dissociating opportunities strictly linked to gender.

## FINAL CONSIDERATIONS

The goal of the authors in this paper was to review the history of women in Science to try to understand the possible causes of gender inequalities in the professional field. One of the questions raised was: if in Brazil, today, there is a significant number of dentists and if women are majority in Dentistry and Orthodontics, why would they not also be majority in the leadership of the profession? What would still prevent women from participating in decision-making centers, at the higher spheres? Are there biological differences between men and women? Or are there specific female limitations and difficulties in understanding and practicing Science? Are these factors due to cultural repression suffered throughout history?

Proportionally to the obstacles observed, the number of women in Science at all times is relatively large, and it would be totally erroneous to think that scientific and technological progress occurred without them.^2^

In the particular case of Dentistry and Orthodontics in Brazil, women’s access to the Academy and to the job market occurred significantly after men, besides being guided by socio-structural issues, many of which are still present today.

There has been great progress and today women are present in all fields of Science, although there is no parity between genders. Since biologically they lead the pregnancy, and socially they are considered responsible for the process of child education and raising, women tend to be marginalized from the productive process, and consequently from strategic occupations. The social structure still considers compulsory motherhood and exclusive dedication as necessary for the scientific career, generating exclusion.^2^ Historical causes and social factors still preclude from perceiving their importance and potential in organizations,^6^ hindering or impeding their progression. There does not seem to be an explicit prejudice, but many men continue to act to guarantee the male hegemony in the highest positions^3^, which is often reinforced by the behavior of women themselves in the way they raise their children or when they do not value the achievements and professional advancement of other women.

Conversely, men should not feel less capable when under the command of a woman. These issues must be faced with professionalism, and leadership must be exercised by meritocracy, regardless of gender. However, when the lowest wages are observed,^2^ ^,^ ^6^ the reduced number of women in leadership positions^2^ and the dedication necessary to reach a certain job, discrimination against women is visible, both in Brazil and abroad. The difficulty of women rising to high positions in organizations is so great that countries like Norway and Sweden have imposed a law on companies that obliges them to reserve a 40% quota for women in fiscal councils.^6^ Initiatives as ongoing educational campaigns in Brazil, which encourage girls to become scientists, as well as programs to discuss unconscious prejudices are necessary.^22^

The indicators presented in this paper should serve as an alert for reflection, since any exclusion can be a form of violence, causing frustration and suffering.^2^ We cannot remain insensitive to the inequalities of our time, not only in the field of Science, but also in how our society is structured. There are many challenges to overcome the “invisibility” of women and this requires awareness of all, especially of women, regarding the change in posture and social structure often favored by themselves, so that the next generations may live in a situation with greater equality of opportunities.

## CONCLUSION

Knowing the history is important in raising the awareness of the persistent socio-structural issue that hides and masks female participation in Science. This reflection and review, based on data collected on the performance of women in Orthodontics in Brazil, can assist in defining the directions that can be followed, modifying the system consciously so that new generations may live in harmony and in a more equal manner. Women are already in a numerical majority in Dentistry and Orthodontics, but more important than that is to act more consistently. The necessary changes in the structure are not only of women and for women, but must involve the entire society, so that rights and duties are distributed equally between genders, while respecting the peculiarities inherent to each person.
